# Prenatal environmental exposures and brain development: studies with baboons and other nonhuman primates

**DOI:** 10.3389/adar.2025.14858

**Published:** 2025-08-11

**Authors:** Igor Y. Iskusnykh, Shiwani Thapa, Victor V. Chizhikov, Anna N. Bukiya

**Affiliations:** ^1^ Department of Anatomy and Neurobiology, University of Tennessee Health Science Center, Memphis, TN, United States; ^2^ Department of Pharmacology, Addiction Science and Toxicology, University of Tennessee Health Science Center, Memphis, TN, United States

**Keywords:** *in utero*, drugs of abuse, alcohol, environmental insult, developmental exposure, neurogenesis, cerebral artery, cerebrovascular development

## Abstract

During pregnancy, the fetal brain undergoes rapid development and is highly sensitive to environmental influences. Understanding the intricate processes that underlie fetal brain development will be critical for advancing maternal-fetal health and mitigating the risks associated with developmental brain disorders. Nonhuman primate (NHP) animal models provide a unique and highly translational platform for studying brain development during pregnancy due to the close anatomical, physiological, and behavioral resemblance of these animals to humans. Our review explores the use of NHP models in elucidating key milestones of prenatal brain maturation and the mechanisms that govern typical and atypical development. We further examine the impact of environmental insults on fetal brain development, including air pollution, infection, ionizing radiation, and exposure to toxicants, and highlight the ways in which these factors can disrupt brain development and neural circuitry, leading to long-term cognitive and behavioral deficits. Recent studies demonstrate that the baboon (*Papio hamadryas*) animal model provides a fruitful yet underused translational model for research related to environmental adverse effects on pregnancy. Lastly, we review the effects of drugs of abuse on the developing fetal brain, highlighting the underlying biological mechanisms identified through clinical and laboratory studies. A combined approach offers a comprehensive understanding of the vulnerabilities of the developing nervous system, informing new strategies for the treatment and prevention of neurodevelopmental disorders.

## Introduction to non-human primate animal model and use of non-human primates in neurodevelopment research

**FIGURE 1:**
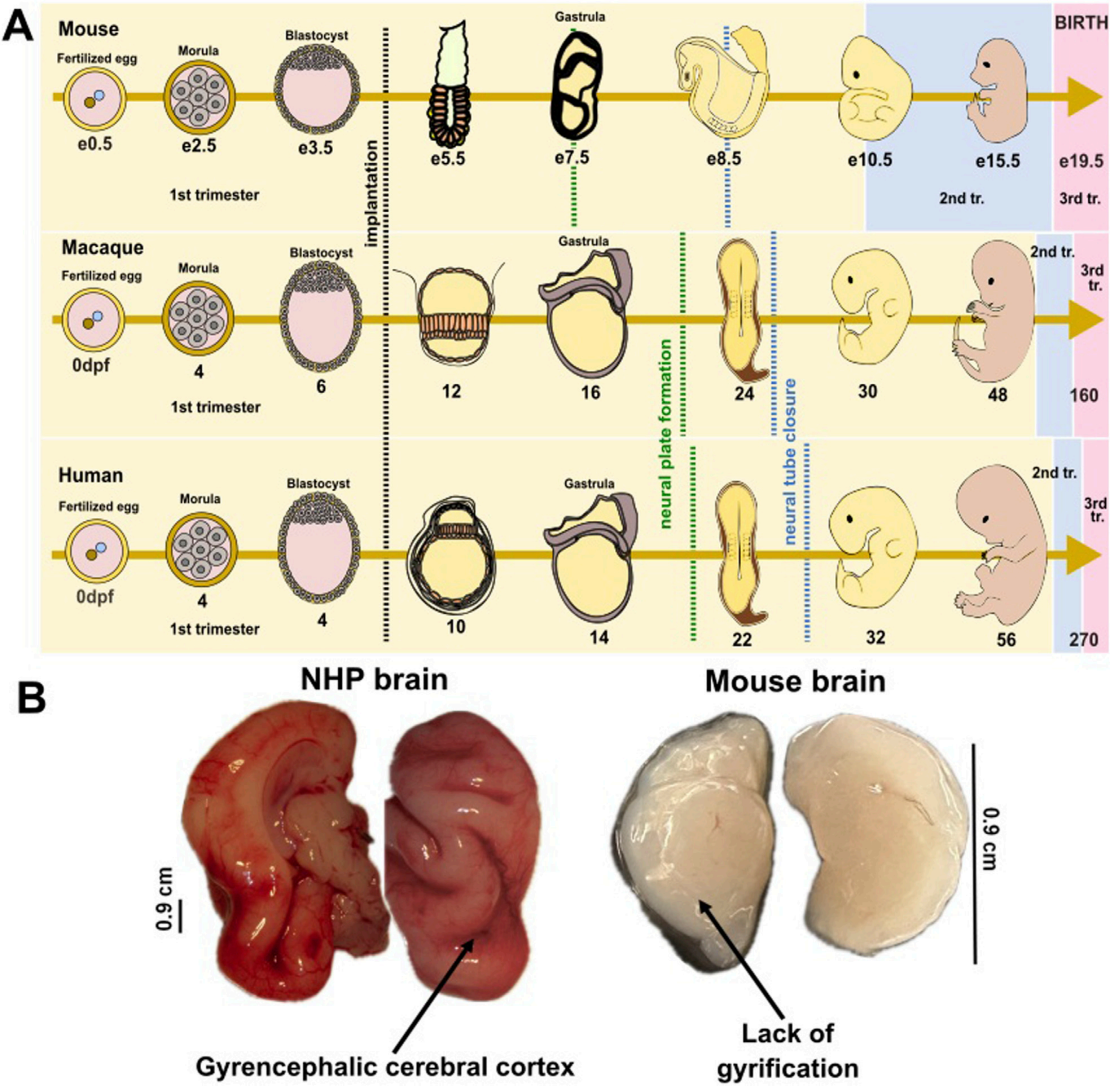
Embryonic and fetal development in mammalian species. **(A)** Schematic illustration of the embryonic development from fertilized egg to morula, blastocyst, and embryo in mice, non-human primates, and humans. While development milestones have not been detailed for baboons, macaque development is well-studied. Crucial developmental processes occur in a similar manner and timing between humans and primates, but the brain’s developmental trajectories show considerable differences between humans and rodents. The black dotted line depicts the period of implantation; the green dotted line depicts the period of neural tube formation; the blue dotted line depicts the period of neural tube closure; e- embryonic day (e0.5 refers to morning after mating, when vaginal plug was detected in mice); dpf- day post-fertilization (refers to number of days since the egg was fertilized). Trimesters of pregnancy depicted by colors: yellow-1st trimester; blue-2nd trimester; pink-3rd trimester. **(B)** Comparative image of fetal brain collected from a near-term (third trimester-equivalent of human pregnancy) NHP (baboon) and from a postnatal day 8 (third trimester-equivalent of human pregnancy) mouse.

A healthy pregnancy supports proper brain development and reduces the risk of neurodevelopmental disorders [1]. The similarities of pregnancy in non-human primates (NHPs) and humans make these animals valuable models for studying human pregnancy and fetal development [2, 3]. Both species experience a similar duration of gestation, with close physiological and hormonal parallels, such as the presence of a placenta that supports fetal growth and the regulation of maternal immune responses to prevent rejection of the fetus [2]. NHPs also undergo similar stages of embryonic and fetal development, including the formation of key brain structures and organ systems (Figure 1A) [3–5].

The 1st, 2nd, and 3rd trimesters across species are shown in Figure 1 using colors (yellow, blue, pink, respectively) [6, 7]. Like humans, NHPs can be affected by environmental factors, infections, or stressors during pregnancy, providing insights into how these exposures influence fetal health and neurodevelopment [2, 4, 8]. These shared characteristics make non-human primates a critical model for investigating human pregnancy-related conditions, fetal development, and the impact of maternal health on offspring outcomes.

NHP have many similarities in developmental processes, physiology, neuroanatomy, reproduction, cognition, and social complexity with humans. [9–11]. As one of the species most closely related to humans, the baboon offers an excellent opportunity for comparative studies of neuronal maturation [12].

Baboons are estimated to share approximately 92% of their genomic sequence with humans [13, 14]. This genetic closeness might determine the similarities between the vascular and neuroanatomical patterns observed in baboon and human populations [15]. Research suggests that baboons exhibit a prolonged infancy and juvenile period, a long lifespan, and complex social behaviour, all of these parallel those of humans, thus rendering these animals ideal for the investigation of cerebral development and associated neurodevelopmental and psychiatric conditions [16]. Baboons and humans share a brain structural organization, including gyrencephalic brain structure, which is indicative of complex neural functions evolved in larger-brained primates [17].This anatomical feature is further illustrated in Figure 1B. Images presented in this figure compare the brains of a baboon and a mouse at similar developmental time-points (third trimester-equivalent of human pregnancy). Images highlight the presence of cortical gyrification in the baboon brain and the absence of such folding in the lissencephalic (smooth) mouse brain.

Brain imaging studies highlight that baboons have a high heritability of brain volume and cortical surface area and display a developmental trajectory in the corpus callosum that closely mirrors that of humans [16, 18] Additionally, both species exhibit similar ratios of grey matter to white matter, which is significant for understanding cognitive processes and potential neurodevelopmental disorders [13] (pre-print) [19, 20]. Importantly, the development of the central nervous system in both species has several similarities. In particular, maturation of the white and grey matter, including gyral formation, myelination, and cortical laminar development, shares strong temporary and structural similarities in baboon and human brains [21]. Baboons are highly suitable for neuroimaging studies due to their large brain size, the highest cerebral gyrification index among common monkeys used in laboratory studies, and the presence of all primary cortical structures homologous to those in humans. [22]. Changes in baboon corpus callosum throughout prenatal (fetal) and postnatal development parallel findings in human neurodevelopment, thereby underscoring the promise of baboons in preclinical models focusing on neurodevelopmental disorders [16]. Baboons and humans also share several key similarities in prenatal development of the cerebellum. Particularly, pronounced similarity in the increase in the thickness of the molecular layer of the cerebellum during the late gestational and early postnatal periods indicate comparable structural and functional development of cerebellar Purkinje cells in the cerebella of both human and baboon [23]. The developing cerebellum undergoes rapid and highly coordinated growth, making it especially vulnerable to various environmental factors affecting its numerous cellular processes.

**FIGURE 2:**
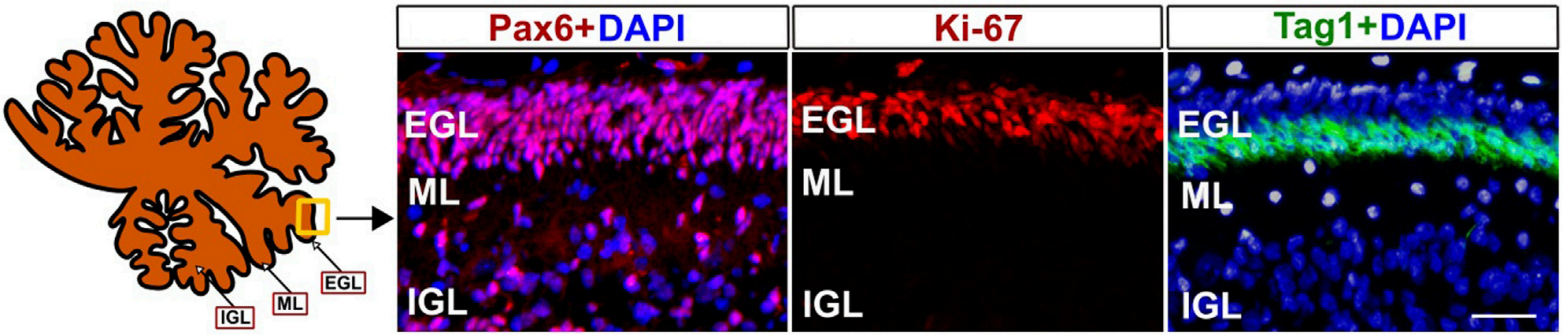
Markers of developing external granular layer (EGL) in baboon cerebellum (120 days of fetal development). Pax6 antibodies mark the progenitors of glutamatergic granule neurons, Ki-67 antibodies mark proliferating progenitor cells, and Tag1+ corresponds to differentiated cells in the inner EGL. Fixation, sectioning, and staining of the Baboon cerebella sections were performed as previously described [24, 26, 27]. Pax6, Ki67, and Taq1 antibodies were used as described in our previous papers [25, 27]. ML- Molecular layer, IGL- internal granular layer. DAPI was used to label cell nuclei. Scale bar - 25 µm.

The external granule cell layer (EGL) is a crucial area of cerebellar development, which, together with the rostral rhombic lip, gives rise to cerebellar granule neurons exclusively. Due to the vital role of the EGL in the differentiation and further migration of granule cell progenitors, changes in cell types may be one of the important mechanisms by which environmental insults induce cognitive and motor impairments [24, 25]. Developing EGL is composed of granule cell progenitors that express *Pax6*. Proliferating granule cell progenitors express *Ki67*. After initiating differentiation, they move to the inner EGL and express *Tag1*. All these cell types are crucial for estimating the development of the EGL in the cerebellum because they represent different cellular states and processes (Figure 2). The abundance of Ki67+ granule cell precursors in the EGL is critical for the production of granule neurons, responsible for motor control, motor learning, and potentially cognitive functions. [24, 25, 28]. The thickness of the Tag1+ layer shows the rate of early neuronal differentiation, indicating the cellular transition from proliferative precursors to migrating neurons. Pax6, Ki67, and Tag1 are markers for granule cell precursors, cell proliferation, and cell differentiation, respectively. Thus, quantification of these cell types may help to identify developmental abnormalities in the cerebellum caused by perinatal environmental insults.

In the developing cerebral cortex, human and non-human primates share an expanded subventricular zone (SVZ) relative to mice. Histological analysis demonstrated that the macaque rhombic lip shows significant similarities with the human rhombic lip, at least at early developmental stages [29]. Developing baboon and human brains exhibit a striking similarity in the temporal decrease in anisotropy relative to other neurodevelopmental stages such as cortical folding and white matter myelination [21]. Since several steps of cerebellar development occur during gestation in humans and baboons, as opposed to postnatally in rodents, the baboon model is particularly important for the study of neurodevelopmental disorders [23]. Particularly, the mechanisms of development of prenatal injury in baboons and humans, such as hippocampal atrophy, loss of cortical grey matter, and particular sensitivity of the subiculum of the hippocampus are highly similar [21].

**FIGURE 3:**
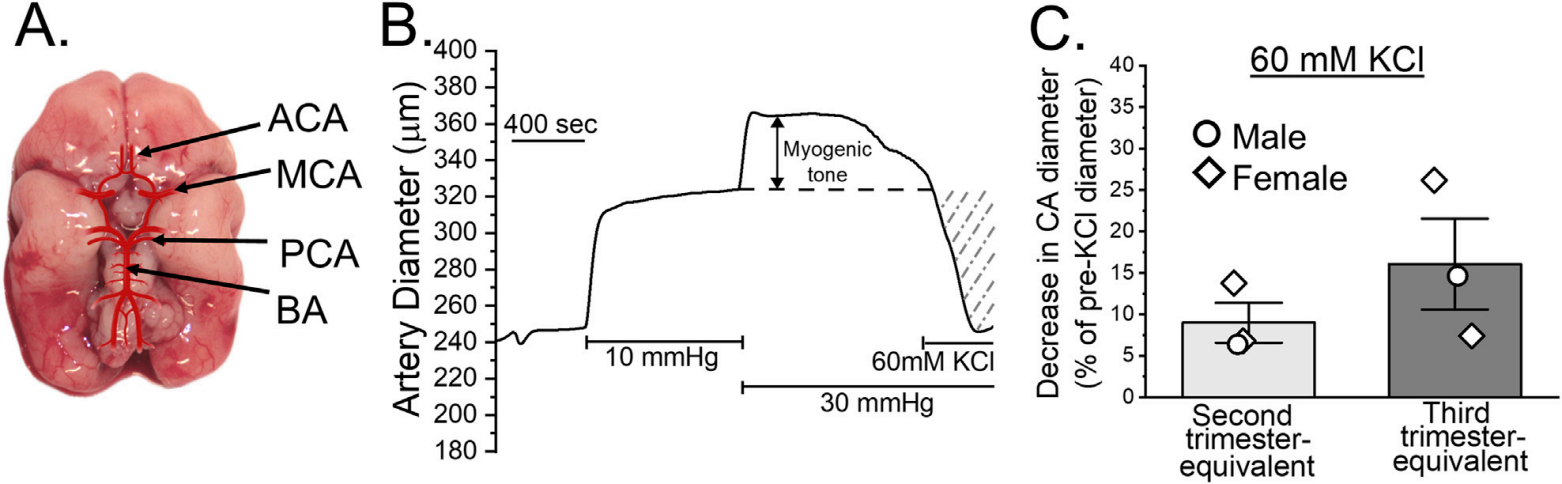
Cerebral arteries in the developing baboon brain. **(A)** Diagram of a fetal baboon brain at gestational day (GD) 120, illustrating the four major cerebral arteries: anterior cerebral artery (ACA), middle cerebral artery (MCA), posterior cerebral artery (PCA), and basilar artery (BA). **(B)** Representative trace showing changes in cerebral artery diameter over time. The artery was dissected out from a male fetus at GD 165, cannulated at both ends and pressurized in an *ex vivo* system. Following a 10-min incubation at 10 mmHg, pressure was increased to 30 mmHg and maintained to assess the development of myogenic tone, as described [30]. **(C)** KCl-induced constriction of fetal baboon cerebral arteries. The y-axis represents the percent change in diameter following exposure to 60 mM KCl compared to a diameter immediately prior to KCl application. Scatter box plots compare arterial responses between baboon fetal arteries collected at the end of the second versus third trimester-equivalent time-points. Circles denote arteries collected from male fetuses; diamonds denote arteries from female fetuses.

In cerebrovascular system, both humans and baboons exhibit similarities in cerebrovascular morphology and responses to various physiological stimuli. Structurally, major cerebral arteries (anterior, middle, posterior and basilar) are already distinguishable in baboon fetuses by gestational day 120, corresponding to the end of the second trimester in humans (Figure 3A). One crucial aspect of cerebrovascular function is the development of myogenic tone. By gestational day 165, equivalent to the third trimester of human pregnancy, baboon fetal cerebral arteries are capable of exhibiting pressure-induced constriction in response to an elevated intraluminal pressure of 30 mmHg (Figure 3B). This indicates presence of a myogenic tone. Moreover, these cerebral arteries also respond robustly to depolarizing stimuli, such as 60 mM KCl, at both the second and third trimester-equivalents of human pregnancy (Figure 3C). Therefore, smooth muscle contractility is also being established during fetal baboon development. Together, these findings show that both the structural formation and functional regulation of cerebral arteries in baboons closely parallel those of humans.

The mechanisms of brain cooling, regulation of blood flow, and adaptation to environmental changes also appear to be analogous in the two species, suggesting a conserved evolutionary pathway for maintaining brain health under duress [31, 32]. Both species possess a comparable vascular architecture, primarily relying on the internal carotid and vertebrobasilar arteries to supply blood to the brain [31, 33]. The anatomy of the carotid system artery, particularly of the internal carotid artery and thoracic aortic arch, and the degree of microvascular collaterals are similar in humans and baboons [34, 35]. The composition of the neurovascular unit, which includes neurons, astrocytes, and endothelial cells, is remarkably conserved across primate species, including humans [36, 37].This unit is vital for neurovascular coupling, ensuring that blood supply meets the metabolic demands of active neurons [37]. The brains of humans and NHPs share similar vascular organization, responses to ischemic episodes, and a cellular environment that supports angiogenesis [38, 39]. The parallels in gene expression and the responsiveness of astrocytes to injury further emphasize the similarities in angiogenic responses between humans and non-human primates [40]. For example, primate-specific responses to stroke, including the roles of astrocytes in modulating macrophage infiltration, illustrate how evolutionary adaptations have shaped the angiogenic process to maintain brain integrity post-injury [39, 40]. The presence of sophisticated inflammatory responses further supports these functional similarities observed in non-human primates and humans [39]. These pathological process such as inflammation, astrocyte activation, etc., accompany many neurodevelopmental disorders [41]. It should be emphasized that the mechanisms of cerebral vascularization and cortical development in NHPs, are markedly different from those seen in rodent models. Subtleties of primate brain vasculature, such as higher vascular density and complexity, suggest a more complex integration of angiogenic signals associated with cerebral growth and functionality, which has implications not only for development but also for the understanding of cognitive capacities unique to primates, thus making primate model superior to rodent model [42]. Non-human primates (NHPs) are characterized by more advanced vascular networks at birth, supporting prolonged development of the cerebral cortex, which includes postnatal neurogenesis and cortical gyrification. In contrast, at birth, rodents exhibit less developed vasculatures that mature postnatally. These differences emphasize the value of NHP models for studying human developmental brain disorders due to their closer vascular and cortical developmental timelines [42, 43].

NHPs demonstrate considerable similarity to humans in terms of cardiovascular physiology and thrombogenic mechanisms. Specifically, baboons offer a significant advantage in research due to the wide availability of cellular markers and advanced non-invasive imaging technologies compared to other large animal models [44]. Moreover, NHP models are used to evaluate the clinical efficacy of existing drugs and other therapeutic interventions. Despite rodents being the most used animal models in biomedical research, treatments shown to be effective in rodent preclinical trials often fail in clinical trials. This may be due to underlying differences between rodents and primates [39]. Thus, the anatomical, structural, and developmental parallels between NHPs and humans reinforce the significance of using baboons in research focused on brain development, genetics, and neurodevelopmental disorders. The similarities in cerebral injury patterns observed in baboon models of preterm birth and neonatal intensive care provide critical insights relevant to human infants that emphasize the high translational relevance of baboon models in understanding the onset and progression of neurodevelopmental disorders. These models allow for a more accurate understanding of how maternal health and environmental toxins can affect fetal neurodevelopment. Environmental insults, such as exposure to pollutants, toxins, viruses, and ionizing radiation, can have devastating consequences on fetal brain development, potentially leading to long-term cognitive, emotional, and behavioral impairments. Research into these environmental factors will help to identify critical windows of vulnerability during pregnancy, offering insights into prevention and intervention strategies to safeguard fetal neurodevelopment and long-term health.

## The impact of environmental insults on pregnancy outcomes: studies with non-human primates address human pathology

Pregnancy is an intricate biological state that allows the fetus to grow and develop via crucial developmental processes. The course and outcome of pregnancy, fetal growth, and development are affected by numerous environmental factors, chemical, physical, and biological. Most importantly, the intake of alcohol, tobacco, and other drugs during pregnancy negatively affects both its course and outcome, as well as fetal growth and development, especially fetal neurodevelopment [45]. Chemical environmental insults that affect fetal development include air pollution, pesticides and herbicides, and heavy metals, the physical environmental insults include radiation and excess heat, and the biological environmental insults include infectious agents, e.g., viruses, bacteria, and parasites [46, 47]. Notably, maternal intake of alcohol, tobacco, cannabinoids, opioids, and other drugs represent modifiable lifestyle factors that can severely affect pregnancy course and outcome, fetal development and growth, and especially developmental trajectory of the brain.

### Chemical pathogens

#### Heavy metals

Exposure to heavy metals in pregnancy can lead to severe adverse outcomes, such as miscarriage, stillbirth, preterm birth, and infants being small for gestational age (SGA) due to their ability to cross the blood-placental barrier [48].

Heavy metal exposure during pregnancy is a significant public health concern due to its potential to cross the placental barrier and adversely affect both maternal and fetal health. Several heavy metals have been implicated in negative pregnancy outcomes and long-term developmental issues in offspring. Arsenic exposure has been strongly associated with miscarriage, stillbirth, infant mortality, and intrauterine growth restriction [48–52]. These outcomes are thought to result from arsenic’s ability to induce oxidative stress, disrupt endocrine function, and impair placental development. Cadmium exposure has been linked to preterm birth and SGA infants [51, 53–55]. Cadmium may interfere with nutrient transport in the placenta and contribute to oxidative damage, which impairs fetal growth and development. Lead remains a critical concern due to its well-documented effects on pregnancy and fetal health. High maternal lead levels have been associated with miscarriage, low birth weight, impaired neurodevelopment, and disrupted bone formation (impaired osteogenesis) in the fetus [53, 56–60].

Mercury exposure during pregnancy has been shown to impair neurodevelopment, particularly affecting cognitive and motor functions in children exposed *in utero* [61]. Methylmercury, commonly found in contaminated seafood, readily crosses the placenta and accumulates in fetal tissues. Manganese isn’t always grouped with heavy metals like lead, mercury, or cadmium, yet it is often considered a heavy metal in discussions about environmental health risks and toxic exposure [62]. Exposure to manganese in excessive concentrations can lead to impaired neurodevelopment [63]. Elevated manganese exposure has been associated with deficits in cognitive function and motor skills in early childhood [64]. Copper deficiency has been previously described in savannah baboons (*Papio cynocephalus*). Copper deficiency can lead to anemia and developmental abnormalities in immature baboons [65]. Importantly, studies on pregnant baboons and their fetuses conducted by Dr. Schlabritz-Loutsevitch and her colleagues analyzed 40 elements using absorption spectrophotometry across multiple biological samples, including maternal and fetal blood, hair, nails, placenta, amniotic fluid, and fetal tissues [66]. Depending on an accumulating organ of the maternal or fetal organism as well as a transport mechanism, elements were found in different concentrations between mother and fetus and between different maternal and fetal organs (including placenta), showing both positive and negative correlation. This study revealed that the amount of these elements in baboons closely mirrored those observed in late-stage human pregnancies. It emphasizes that pregnant baboons serve as a valuable model for studying both normal maternal-fetal physiology and environmental toxicology. This research advances the medical field by providing a non-human primate model that closely parallels human pregnancy, thereby facilitating a better understanding of nutrient and toxin transfer during gestation.

#### Anesthetic-induced developmental neurotoxicity

Due to neurodevelopmental similarities to humans, nonhuman primate models offer a valuable translational tool for studying anesthesia-induced developmental neurotoxicity. NHP models allow researchers to investigate the effects of anesthetic exposure on brain development, revealing long-term behavioral and cognitive impairments. Recent studies in primates have demonstrated that early exposure to anesthetics can lead to an increase in anxiety-like and inhibition behaviors. Moreover, histopathological analysis of NHPs’ brains revealed that exposure to isoflurane during infancy led to increased astrogliosis 2 years after the exposure, indicating chronic astrocyte activation [67]. These studies offer critical insights into the mechanisms and long-term outcomes relevant to pediatric anesthesia safety.

#### Pesticides and herbicides

Exposure to pesticides and herbicides in pregnancy can lead to severe adverse outcomes, such as miscarriage, stillbirth, preterm birth, and birth defects [68, 69].

Importantly, even preconception exposure to certain pesticides has been associated with an increased risk of stillbirth. Specifically, pesticides linked to stillbirth risk during the preconception period include zeta-cypermethrin, organophosphates, malathion, cyfluthrin, and carbaryl [68, 69]. Similarly, exposure to certain pesticides during the first trimester of pregnancy has also been associated with stillbirth. These include fenpropathrin, permethrin, organophosphates, acephate, and formetanate hydrochloride [68, 69]. The toxicity mechanisms of these pesticides are largely unknown; however, the metabolites of permethrin and cypermethrin, which are also shared with zeta-cypermethrin, interact with cellular estrogen receptors, affecting women’s reproductive cycles, altering cycle lengths, and impacting the overall quality of the uterine environment during pre-implantation. Exposure to herbicides (glyphosate) is associated with shortened pregnancy lengths and reduced fetal growth [68–70]. Moreover, it affects neurodevelopment, as it was demonstrated in rats [69, 71]. A prolonged exposure to pesticides of Pigtail macaques (*Macaca nemestrina*) during pregnancy potentially caused a significantly increased level of infant mortality (more then 50%) compared to 30% infant mortality in the pigtail macaques groups habituating in areas not affected by pesticides [72]. Moreover, prenatal pesticides of baboons and chimpanzees caused congenital deformities, including cleft palate, as well as abnormal pigmentation and lowered fertility [73].

### Physical pathogens

#### Ionizing radiation

Radiation exposure in pregnancy can lead to severe adverse outcomes, such as miscarriage, stillbirth, growth restriction, abnormal development, especially impaired neurodevelopment, malformations, and, most importantly, mutagenesis and carcinogenesis. The risk and type of such consequences depend largely on the stage of fetal development (dpf/embryonic day/gestational day) and the radiation dose. Both embryo and fetus are most sensitive to ionizing radiation at doses greater than 0.1 Gy (Gy). Even lower acute ionizing radiation doses can adversely affect both embryonal and fetal development. Dosage 0.05–0.5 Gy at 0–2 weeks post-conception may affect implantation of the embryo, and at 2–7 weeks post-conception may slightly affect organogenesis of the embryo. At early stages of fetal development (8–15 weeks), such dosage can cause growth restriction and lowered IQ in the future. Dosage higher than 0.5 Gy may cause miscarriage at any stage of embryonal and fetal development and even neonatal death at 38th postnatal week. Moreover, there is a significantly high risk of growth restriction, severe malformations, and lowered cognitive function. Higher doses of acute ionizing radiation prenatal exposure (1–5 Gy) are considered lethal [74–78].

Despite the rarity of environmental radiation exposure, pregnant women still frequently encounter various forms of ionizing (x-rays, computed tomography) and non-ionizing (ultrasound, magnetic resonance imaging) exposures in the form of medical imaging. Some medical procedures and imaging techniques exposing patients to low doses of radiation are generally considered safe. Yet, special considerations still must be made for their use in pregnancy to ensure the optimal development of the fetus and maternal health [79, 80]. The baboon animal model has been used to investigate biomarkers associated with radiation exposure [81]. The authors aimed to identify biomarkers that distinguish total-body irradiation and partial-body irradiation. Interestingly, the key biomarkers found included aspartate aminotransferase, LDH, urea, Flt3-ligand, iron, creatine kinase, absolute neutrophil count and neutrophil-to-lymphocyte ratio for the early period after the radiation exposure, C-reactive protein, and Flt3-ligand, platelet count, iron, hemoglobin, monocyte count, absolute neutrophil count and neutrophil-to-lymphocyte ratio for the acute radiation syndrome phase. In the study, biomarkers such as aspartate aminotransferase, LDH, Flt3-ligand, and neutrophil-to-lymphocyte ratio were identified as early indicators of tissue damage and hematopoietic stress caused by radiation. During the acute radiation syndrome phase, biomarkers like C-reactive protein, platelet count, and hemoglobin reflected systemic inflammation and hematopoietic impairment. These biomarkers help distinguish the extent of radiation exposure and the design of treatment protocols.

Thus, this data obtained using baboons as a clinically relevant animal model can be integrated into diagnostic and prognostic strategies to improve medical care for individuals exposed to ionizing radiation.

#### Air pollution

Air around the world is polluted by multiple sources. There is gaseous pollution with increased concentrations of greenhouse gases such as carbon dioxide (CO_2_), methane (CH_4_), nitrous oxide (N_2_O), ozone (O_3_), and fluorinated gases formed from the burning of fossil fuels. Particulate matter pollution is a mixture of various solids and aerosols, including particles of metals, dust, soil or dust particles, allergens, and numerous chemicals, both synthetic and natural. The diameter of particles being less than 10 μm (PM10) or, most importantly, less than 2.5 μm (PM2.5) is associated with adverse outcomes, depositing in the airways, lungs, or even entering the circulatory system. Critically important is indoor air pollution, which consists of particles of allergens such as mold spores and dust mites, smoke from cigarettes and burning stoves, and volatile chemicals originating in house cleaning products. Exposure to air pollution in pregnancy is associated with adverse pregnancy outcomes such as an increased risk of preterm birth, low birth weight (SGA), increased neonatal mortality, stillbirth, and miscarriage [82–84].

#### Excess heat

Excessive heat exposure during pregnancy can lead to adverse outcomes, including preterm birth, stillbirth, low birth weight, and congenital abnormalities (such as heart defects, neural tube defects, and ocular development defects) [85]. Using pregnant baboons as an NHP animal model it was demonstrated that maternal hyperthermia described as an absence of fever with body temperature 41–42°C caused a blood pressure drop and an elevation in heart rate in the fetus as well as severe acidosis (blood pH less than 7.2), hypoxia and hypercapnia (partial pressure of carbon dioxide in blood above 45 mmHg) [86, 87]. Moreover, excess heat exposure of the pregnant baboons causes an increased uterine contractility up to two-fold of the normal level, which may lead to a preterm birth [87, 88]. Even a single day of high heat can elevate the risk of pregnancy complications [85, 89–93].

### Microbiology pathogens

#### Viral infection in pregnancy

The ubiquity of infectious diseases in pregnancy makes this biological insult a notable special consideration in healthcare. Viral infection in pregnancy can lead to increased maternal morbidity and mortality, miscarriage, stillbirth, intrauterine growth restriction (IUGR), and severe birth defects (e.g., microcephalia), congenital infection. Viruses can infect the decidua and placenta or directly infect the fetus [94, 95]. Table 1 represents studies describing the effects of various viruses on pregnancy and fetal development, including viruses such as CMV, Zika (ZIKV), and Rubella (RuV), which cause congenital infections and severe outcomes like miscarriage, stillbirth, and IUGR.

**TABLE 1:** Adverse effects of viral infection on fetal development and pregnancy outcome in human population.

Virus	Abbreviation	Virus family	Route of infection, and mode of effect	Effect on pregnancy and fetus	References
Cytomegalovirus	CMV	*Herpesviridae*	Intrauterine transmission	Congenital infection, abnormal neurodevelopment, microcephaly, impaired development of hearing and vision, stillbirth, IUGR, preterm birth (human) Spontaneous abortions (macaque)	[94–99]
Herpes simplex virus 1–2	HSV-1 HSV-2	*Herpesviridae*	Infects decidua, placenta causing systemic and local changes	Miscarriage, stillbirth, abnormal neurodevelopment, congenital disease, preterm birth	[94–96, 100]
Human papilloma virus	HPV	*Papillomaviri-dae*	Infects placenta	Preterm birth, miscarriage, IUGR, stillbirth	[94, 101, 102]
Zika virus	ZIKV	*Flaviviridae*	Infects decidua, placenta causing systemic and local changes	Congenital infection, Severe developmental abnormalities: Fetal brain sequence (severe microcephalia, premature closure of fontanels, partial scull collapse), brain abnormalities, ocular abnormalities, IUGR with “femur-sparing” profile Miscarriage, preterm birth, stillbirth (human) Miscarriage (monkey)	[94, 95, 100, 103]
Rubella virus (*Rubivirus rubellae*)	RuV	*Matonaviridae*	Transplacental infection	Congenital infection (rare), abnormal neurodevelopment Miscarriage, premature birth, IUGR, congenital rubella syndrome (triade: cataracts, congenital heart defects, deafness) (human) Spontaneous abortion, fetal lesions, IUGR (monkey)	[94, 96, 104–106]
Influenza virus A	IAV	*Orthomyxoviridae*	Maternal systemic infection	Preterm birth, miscarriage, IUGR, birth defects such as cleft palate, neural tube defects, congenital heart defects (Human) Influenza infection during pregnancy affects neural development, reducing gray and white matter (monkey)	[94, 107–109]
Severe acute respiratory syndrome coronavirus 2	SARS-CoV-2	*Coronaviridae*	Possible transplacental infection, maternal systemic infection	Congenital infection, stillbirth	[100, 110, 111]
Hepatitis A, B, C, and E viruses	HAV HBV HCV HEV	*Hepadnaviri-dae*	HBV, HCV, HDV- maternal liver disease consequences HBV, HCV- mother-to-child transmission Transplacental infection	HBV-preterm birth, low birth weight HAV-preterm birth HEV- severe fetal hepatitis, stillbirth, preterm birth, low-birth weight HCV-IUFGR, low birth weight, stillbirth, preterm birth (human) Premature delivery and fetal death (HEV) (macaque)	[94, 100, 112–118]
Human Immunodeficiency Virus	HIV	*Retroviridae*	Mother-to-child transmission	Untreated maternal HIV-premature birth, SGA, low birth weight, stillbirth, abnormal neurodevelopment (human) Developmental delay (macaque)	[100, 119–122]
Varicella zoster virus	VZV	*Herpesviridae*	Vertical transmission	Fetal varicella syndrome (cutaneous scars, limb defects, eye and brain abnormalities), IUGR	[96, 123–125]

As was demonstrated in research conducted on baboons (*Papio hamadryas*), viral infection with herpesvirus papio 2 (HVP2) and cytomegalovirus (CMV) affects the placenta and causes placentitis, leading to adverse effects in pregnancy with a high prevalence in the baboon population up to 95% seropositivity [96]. Notably, the baboon model was successfully used for testing the placental transfer and fetal metabolism of antiretroviral drugs such as zidovudine (used in pregnancy to lower maternal-fetal HIV transmission). By using pregnant baboon dams, it was demonstrated that zidovudine and its glucuronide metabolite were able to cross the placenta, with evidence of fetal metabolism [126]. Moreover, baboons have been used in virology research to study the effects of the Zika virus infection on fetal development. Importantly, this study demonstrated that perinatal Zika virus infection can cause a significant fetal cerebral cortical injury resulting in fetal death in baboons, underscoring the baboon’s high value as an animal model of pregnancy and perinatal viral infection affecting fetal neurodevelopment [127].

#### Bacterial infection in pregnancy

Bacterial infection in pregnancy can cause fetal congenital disease, miscarriage, stillbirth, chorioamnionitis (inflammation of the fetal membranes), preterm birth, and low birth weight. Oral, sexually transmitted, or commensal bacterial infection in pregnancy can be transmitted vertically to the fetus and impact its development as well as pregnancy outcome. Bacterial vaginosis, caused by multiple bacteria, can also lead to premature labor and birth [128, 129]. Table 2 outlines the impact of various bacterial infections on fetal development and pregnancy. These infections are divided into categories: sexually transmitted infections (STIs), commensal bacteria, and those acquired through contaminated food or animal contact. This helps to emphasize the need for screening, early treatment, and preventive measures like food safety to mitigate negative outcomes on pregnancy. As was demonstrated by research with baboons (*Papio hamadryas*), bacterial infection with *Ureaplasma urealyticum* and *Klebsiella spp*. caused placentitis and intrauterine infection, leading to severe adverse effects on pregnancy, such as stillbirth and intrauterine growth restriction [96].

**TABLE 2:** Adverse effects of bacterial infection on fetal development and pregnancy outcome in human population.

Type of infection	Bacteria	Effects on pregnancy and fetus	References
STI	*Chlamydia trachomatis*	Preterm birth, congenital infection (human) Births of weak, low-weight, and vitality-monkey calves were observed in infected macaques	[96, 129–132]
	*Neisseria gonorrhoeae*	Low birth weight, preterm birth	[129, 131]
	*Treponema pallidum*	Stillbirth, miscarriage, low birth weight	[96, 129]
	*Trichomonas vaginalis*	Preterm birth, low birth weight	[129, 131, 132]
	*Ureaplasma urealyticum*	Preterm birth, low birth weight	[96, 129, 132]
	*Mycoplasma hominis*	Preterm birth, low birth weight	[96, 129, 132]
Commensal	*E. coli* (bacterial vaginosis)	Preterm birth, stillbirth	[96, 129]
	Group B *streptococcus*	Preterm birth	[129, 133]
Contaminated food consumption/contact with an infected animal	*Listeria monocytogenes*	Congenital disease, stillbirth, miscarriage (human) Stillbirth (monkey)	[129, 134, 135]
	*Brucella spp*	Miscarriage, preterm birth, chorioamnionitis	[129]

#### Endoparasitic infection in pregnancy

Internal parasitic infection in pregnancy includes protozoan and helminth infection. Helminth infection, amebiasis can affect pregnancy outcome via systemic maternal adverse effects such as malnutrition and anemia. Infections such as malaria (caused by plasmodium) and amebiasis can cause preterm birth in humans [136, 137]. Notably, the protozoa *Toxoplasma gondii* causes toxoplasmosis, which has severe consequences on pregnancy and fetal development due to vertical transmission of the infection, causing severe developmental abnormalities of the neurodevelopment and both ocular and cardiac systems [96, 138].

The most important and severe biological environmental insults are separated into the acronym ToRCH, describing the most notable pathogens causing severe perinatal infection negatively affecting the fetus and pregnancy outcome. ToRCH stands for toxoplasmosis, other (*Treponema pallidum*, hepatitis viruses, HIV, varicella, parvovirus B19, enteroviruses, Zika virus, Dengue, MERS, SARS, SARS-CoV-2), rubella, cytomegalovirus, and herpes simplex virus [139–142]. ToRCH infections are severe, and some of these pathogens can be transmitted from mother to fetus and cause severe congenital abnormalities. In case the pathogen is unable to cross the placenta, it still may severely affect maternal health to the point of negative consequences for the fetus [143].

### Psychoactive substances

#### Alcohol (ethyl alcohol)

Ethanol exposure during prenatal development has been extensively studied using NHP models, including rhesus and vervet monkeys and notably baboons [144, 145] due to their close genetic and neuroanatomical resemblance of humans and the similarities in the developmental processes during pregnancy. These studies have provided critical insights into how ethanol affects neurodevelopment, mirroring aspects of human fetal alcohol spectrum disorders (FASD) [146]. FASD includes a spectrum of physical, cognitive, behavioral and neurodevelopmental impairments. Notably, the developing brain is the most vulnerable to ethanol toxicity organ [147, 148].

To closely mimic human FASD, researchers have developed NHP models where pregnant rhesus macaques voluntarily consume ethanol. These models take advantage of the similarities between humans and rhesus macaques in gestational length relative to brain development, as well as similarities in ethanol self-administration and metabolism [149–151]. Studies using this model have shown that a daily ethanol dose of 1.5 g/kg during the first trimester does not influence pregnancy success rates but does affect drinking behavior during the second month of pregnancy [149]. Subsequent research using this model aims to describe the effect of early-gestation ethanol exposure on anatomical and functional brain development at different gestational ages [149, 150, 152].

The timing of prenatal ethanol exposure plays a crucial role in determining its neurodevelopmental outcomes. In a study, rhesus monkeys were exposed to moderate amounts of ethanol (0.6 g/kg, voluntary alcohol consumption daily) during different gestational periods, namely, early gestation (gestational days 0–50), mid-to-late gestation (gestational days 50–135) or continuous exposure throughout gestation [151]. This exposure led to blood alcohol levels ranging from 20 mg to 50 mg/dL (∼4.3 mM–11 mM). The early gestation exposure to alcohol significantly reduced neurodevelopmental tests scores, including diminished infant orientation and motor maturity. In contrast, mid-to-late gestation exposure primarily affected motor maturity. These results obtained in non-human primates, underscored the heightened sensitivity of early gestational periods to the neurotoxic effects of ethanol [151]. During the first trimester of pregnancy, rhesus monkeys exposed to ethanol exhibited reduced placental blood flow, decreased overall growth, and impaired growth and development of the brain [153]. In baboons, fetal growth restriction has been documented following three episodes of maternal intragastric gavage with alcohol during the second trimester-equivalent of human pregnancy. Maternal blood alcohol levels were around 80 mg/dL, while the alcohol level in the amniotic fluid reached 63 mg/dL [30]. While 80 mg/dL (∼17 mM) represents the legal blood alcohol concentration limit for driving a motor vehicle in most of the United States, a concentration of 63 mg/dL (∼13.7 mM) marks the onset of noticeable behavioral impairment in humans [154, 155]. Near term, baboon progeny that was exposed to alcohol during the second trimester-equivalent had reduced circumferences of the abdomen and head without affecting femur length [145].

Besides growth curves, prenatal alcohol exposure has been documented to negatively impact neurogenesis and neurotransmitter systems in the developing brain. In vervet monkeys, exposure to ethanol, ranging from 13 mM to 29 mM, during the third trimester led to reduced numbers of hippocampal neurons and loss of hippocampal volume [156].

Moderate prenatal exposure of rhesus monkeys to alcohol, combined with serotonin transporter gene promoter (rh5-HTTLPR) polymorphism, altered the function of serotonin in the central nervous system. This exposure led to maternal blood alcohol levels of 20 mg–50 mg/dL (∼4.3 mM–11 mM). Monkeys carrying the short allele of the rh5-HTTLPR exhibited reduced cerebrospinal fluid levels of the serotonin metabolite 5-hydroxyindoleacetic acid (5-HIAA), suggesting a gene-environment interaction that may contribute to neurodevelopmental impairments associated with prenatal alcohol exposure [157].

Epigenetic mechanisms, including DNA methylation and histone modifications, have been implicated in the neurodevelopmental abnormalities that result from prenatal ethanol exposure. Temporal lobe samples from the brains of both humans and NHPS (macaque) with documented prenatal alcohol exposure exhibited significant decreases in global methylation of DNA and histones and increased histone acetylation, indicating that prenatal alcohol exposure can lead to widespread epigenetic changes that may underlie neurodevelopmental deficits [152].

Prenatal ethanol exposure also affects both gene expression and regulation by microRNAs (miRNAs) in the developing brain. During neurodevelopment, ethanol exposure disrupts miRNA expression, altering the regulation of genes which are critical for synaptic development, as well as proliferation, and migration of neuronal progenitors. Thus, ethanol-induced miRNA expression disruption results in structural and functional brain developmental defects [158]. In vervet monkeys, exposure to ethanol during the last 2 months of gestation led to reduced numbers of cortical and hippocampal neurons, accompanied by upregulation of miRNAs in the hippocampus [146]. It was significantly correlated with reduced expression of their predicted targets-messenger RNA (mRNA), which miRNA typically bind at the 3′ untranslated region, inducing mRNA degradation or inhibiting their translation. mRNAs are responsible for the biosynthesis of key proteins involved in developmental processes such as migration, differentiation, and proliferation. As it was observed in a previous study by Gillis et al. [150], mRNA was globally downregulated in vervet monkeys prenatally exposed to ethanol; thus, these results suggest that ethanol-induced upregulation of specific miRNAs may contribute to the downregulation of expression, potentially leading to neurodevelopmental impairments. Interestingly, these studies uncovered a previously unknown link between FASDs and the *EFNB1* gene that encodes ephrin B1, which plays a crucial role in neurodevelopment and the development of craniofrontonasal syndrome. Considering the functions of *EFNB1*, this novel connection suggests a significant area for further investigation into the etiology and potential therapeutic targets of FASD [146, 150].

Computational models have been utilized to predict ethanol-induced neurodevelopmental toxicity across species. One such model applied mechanistic data from rodent studies to evaluate the sensitivity of primate species to ethanol-induced inhibition of neocortical neuronal proliferation. The model predicted that primates, including humans, are more sensitive to ethanol’s effects, with significant neuronal deficits occurring at lower blood ethanol concentrations compared to rodents. For example, the model predicted a significant decrease in neocortical neuronal number after prenatal ethanol exposure in a dose simulating a consumption of one standard drink within 1 hour in human. This increased sensitivity is attributed to the prolonged rapid growth period, compare to rodents, in the primate neuronal progenitor population highlighting the relevance of NHP models in assessing ethanol’s impact on human neurodevelopment [159].

The development of baboon model of FASD has provided valuable insights into the effects of prenatal alcohol exposure on primate development. Baboons (*Papio* spp.) are particularly suitable for FASD research because of their close genetic, anatomical, and physiological similarities to humans. Their complex brain structure, prolonged gestational period (approximately 6 months), and advanced social and cognitive behaviors make them an excellent model for studying the neurodevelopmental, behavioral, and physical consequences of alcohol exposure during pregnancy [160].

Approaches used in FASD studies using baboons as a large animal model typically involve administering ethanol to pregnant females through controlled dosing, which replicates human drinking or gastric infusion to maintain stable blood alcohol levels [30, 161]. One study reported administration of ethanol to baboon imitating a single binge drinking episode in an adult human. Ethanol was administered to the pregnant baboon dam via a gastro-nasal catheter at a dose of 3 gm of ethanol per kg of weight to approximate a blood-alcohol level concentration of ∼0.2%. This dose of alcohol is equivalent to the consumption of 6–8 alcoholic drinks in 2 hours by an adult human imitating a heavy binge-drinking episode [162]. The timing of fetal alcohol exposure is carefully managed to correspond to critical periods of human prenatal brain development, particularly during the first and second trimesters, when the brain is most vulnerable. Vasoactive properties of alcohol caused an increased placental permeability as well as an increased fetal brain perfusion making fetal brain more vulnerable to toxic insults [162].

In addition to its effects on neurodevelopment, prenatal alcohol exposure also compromises the fetal cerebral circulation [30, 145, 162–165]. Using the baboon model, a study from the Bukiya lab showed that moderate doses of ethanol (∼13.7 mM) administered during the second trimester of pregnancy can induce significant dilation of the middle cerebral artery, indicating disrupted regulation of vascular tone [30]. This period represents a critical window for cerebrovascular development [166, 167]. A subsequent study from the Bukiya lab implicated the endocannabinoid system in mediating this effect. Bukiya et al demonstrated that a pharmacological block of cannabinoid receptors CB1 and CB2 effectively reversed the ethanol-induced vasodilation [145]. Furthermore, proteomic profiling of cerebral arteries collected in the third trimester from baboon fetuses exposed to ethanol during mid-gestation revealed long-term molecular alterations, with the most prominent changes observed in mitochondrial and cytoskeletal proteins [144]. Such alterations may impair mitochondrial function, alter fetal cerebral artery contractility, and compromise vascular integrity. Collectively, these studies suggest that prenatal ethanol exposure disrupts both the structural and functional development of fetal cerebral arteries. However, the specific molecular mechanisms, receptor-specific contributions and long-term cerebrovascular consequences remain to be fully elucidated.

Advancements in neuroimaging and molecular biology will further enhance the understanding of the mechanisms by which alcohol disrupts fetal development in primates and may guide the development of targeted prevention and treatment strategies for FASD in humans [168] Owing to the long lifespan [169], baboons offer promising opportunities for longitudinal studies that can track the long-term effects of prenatal alcohol exposure and interventional studies to mitigate the impacts of FASD.

#### Cannabinoids

The legalization of marijuana is leading to an increased belief in the “safety” of the product. It led to a consequent growth of cannabis misuse in the population, including in pregnancy [170]. The most prevalent form of drug misuse during pregnancy is the maternal use of cannabinoids, with approximately 5% of pregnant women self-medicating with marijuana [171]. Cannabinoids, including tetrahydrocannabinol (THC) and cannabidiol (CBD), have been increasingly studied for their effects on neurodevelopment. NHP models provide critical insights into the long-term impact of cannabinoid exposure due to their close genetic and neuroanatomical similarity to humans [172, 173], and highlight the effects of perinatal maternal cannabinoid use, which may cause preterm birth and low birth weight [174].

The endocannabinoid system plays a crucial role in neurodevelopment, regulating synaptogenesis, neuronal differentiation, and circuit maturation [175]. Exogenous cannabinoids, including THC and CBD, can disrupt fetal endocannabinoid system signaling, potentially leading to long-term neurodevelopmental consequences [176, 177]. Given the ethical and methodological limitations of human studies, NHP models provide valuable insights into the effects of prenatal cannabinoid exposure on cognitive function and behavior [178].

Prenatal exposure to cannabinoids, particularly THC, has been linked to alterations in brain morphology [174] and functional connectivity in humans [179]. Chronic prenatal exposure to delta-9-tetrahydrocannabinol (THC) in a rhesus macaque model resulted in significant alterations in fetal brain development. MRI assessments revealed that THC exposure affected brain growth in both male and female fetuses compared to controls. Histological analysis at gestational day 155 indicated signs of brain dysregulation in the THC group. Additionally, two extracellular vesicle-associated microRNAs were identified in the cerebrospinal fluid of THC-exposed fetuses, with pathway analysis suggesting disrupted axonal guidance and netrin signaling [180].

**FIGURE 4:**
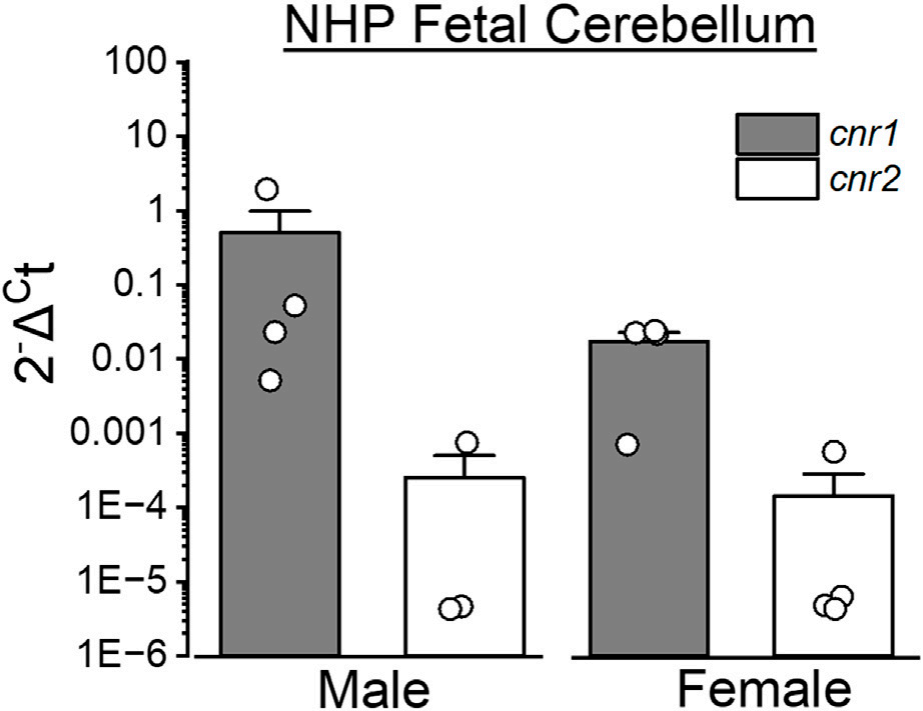
Expression of cannabinoid receptors-encoding genes in the fetal cerebellum of baboons. Cerebellar tissue was collected from male and female baboon fetuses at gestational day 120 (end of second trimester-equivalent of human pregnancy) to assess the expression level of cannabinoid receptor-encoding genes (*Cnr1* and *Cnr2* encoding CB1 and CB2 receptors, respectively). Quantitative PCR (qPCR) was performed using TaqMan Gene Expression Assays (ThermoFisher Scientific). The y-axis represents relative gene expression, where Ct is the cycle number at which fluorescence surpasses the detection threshold. Expression of *Cnr1* and *Cnr2* was normalized to β-actin (Actb) in each sample. Probes used: *Cnr1* (Rh02787040_s1), *Cnr2* (Rh02913156_m1) and *Actb* (Rh02621734_g1). qPCR procedures were performed using previously published protocols [181]. Sample sizes were n = 4, except for *Cnr2* in male fetuses (n = 3). Each sample was collected from a separate baboon fetus.

Cannabinoids exert their effects primarily through CB1 and CB2 receptors, which are abundantly expressed in the developing brain. As shown in Figure 4, both CB1 and CB2 transcripts are detectable in the cerebellum of fetal baboons.

As a partial agonist at CB1 receptors, THC disrupts the tightly regulated balance of excitatory and inhibitory neurotransmission, leading to long-term synaptic deficits [182, 183]. Additionally, epigenetic modifications, including DNA methylation and histone modifications, have been observed after prenatal cannabinoid exposure, suggesting that effects may persist in the next-generation (intergenerational effect) or even across multiple generations (transgenerational effect) [184, 185]. Cannabis exposure during critical developmental windows can disrupt epigenetic processes, leading to heritable changes in genes and molecular pathways. These alterations are linked to psychiatric diseases like autism spectrum disorder, attention-deficit/hyperactivity disorder (ADHD), schizophrenia, and addiction. Functionally, prenatal cannabinoid exposure has been associated with deficits in executive function, aggression, increased impulsivity, altered social behaviors, and other psychiatric disorders [186, 187]. Thus, increased cannabis use, especially during brain development, has been associated with a rise in mental health issues among adolescents and young adults in the U.S. [187]. Primate models provide critical insights into the neurodevelopmental impact of cannabinoid exposure, highlighting structural, functional, and behavioral consequences. Under-utilization of NHPs, including baboons, in research may impede further progress in elucidating dose-dependent effects, the role of genetic predisposition, and potential interventions to mitigate adverse outcomes of developmental exposure to cannabis.

#### Tobacco

The impact on fetal neurodevelopment of tobacco exposure during prenatal and perinatal periods has been extensively studied in NHPs, particularly rhesus monkeys [188]. Rodents such as rats and mice are altricial species, so their brain development at birth corresponds to fetal stages of human development; therefore, the concentrations of nicotine may not be correlated with those observed in typical human exposure scenarios [189].

Studies involving rhesus monkeys exposed to tobacco both as direct nicotine exposure and as environmental tobacco smoke (ETS) during the perinatal period have demonstrated significant alterations in brain development. Perinatal exposure to ETS in rhesus monkeys resulted in selective upregulation of nicotinic acetylcholine receptors in the brainstem and cerebral cortex. This change was selective, with no effects on m [2]-muscarinic or beta-adrenergic receptors. The upregulation of nicotinic receptors suggests chronic nicotine stimulation, a hallmark of nicotine-induced neuroteratogenesis, indicating that perinatal ETS exposes the fetus and neonate to nicotine levels that can alter brain development [189, 190].

ETS (also known as involuntary, secondary, or passive smoking) causes multiple adverse effects on exposed subjects. In particular, prenatal ETS exposure leads to region-specific neurodevelopmental damage in primate brains. The neurotoxicity and neuroteratogenic effects of nicotine exposure disrupt the formation, survival, and differentiation of brain cells, leading to apoptosis and reduced cell size in the forebrain, midbrain, and hindbrain. These lead, in turn, to structural deficits, impaired synaptic function, and behavioral abnormalities in rats [191, 192]. These findings underscore the vulnerability of the developing primate brain to nicotine and highlight the potential for long-term neurodevelopmental impairments [193].

Bruin et al have examined the enduring effects of fetal and neonatal nicotine exposure on postnatal health, emphasizing the increased risk for neurodevelopmental disorders such as ADHD, anxiety, and depression. These outcomes are linked to alterations in brain regions like the prefrontal cortex, and hippocampus, and changes in neurotransmitter systems, including nicotinic acetylcholine receptors [194].

The parallels between findings in NHP studies and human epidemiological data are striking. Prenatal tobacco exposure in humans is associated with an increased risk of behavioral disorders in children and adolescents, not only ADHD, but oppositional defiant disorder, and conduct disorder. Studies in NHPs have been crucial in elucidating the detrimental effects of tobacco exposure on neurodevelopment. They pave the way to the development of new strategies of a neurodevelopmental disorders treatment and prevention.

## Discussion, concluding remarks and future directions

Despite the scientific advantages of using NHPs, their enrollment into research entails significant ethical and logistical challenges. Ethical considerations are paramount in primate research, necessitating strict adherence to welfare regulations and a clear justification for the use of NHPs [168]. Moreover, many NHPs (including baboons) have long lifespans and require complex care, making these studies expensive and resource-intensive. Despite these challenges, we believe that the use of NHPs in research is beneficial. NPH is a unique translation model for human diseases and conditions. NHPs have a complex brain, allowing them to perform sophisticated behavioral, visual, and electrophysiological studies for elucidating mechanisms and new treatment strategies of human neurodevelopmental disorders such as ADHD, autism spectrum disorders, etc. Environmental insults adversely affecting pregnancy and fetal development, especially neurodevelopment, are ubiquitous and numerous, and, as demonstrated in previous research [160, 195], the NHP animal model provided by the baboon *Papio hamadryas* is a great translational model for the research of pregnancy, placental, and fetal development. The insights gained from NHP studies are crucial for advancing preventive and therapeutic strategies to mitigate the impact of environmental exposures on human development.

## Abbreviations

ADHD, attention-deficit/hyperactivity disorder; CBD, cannabidiol; EGL, external granular layer; ETS, environmental tobacco smoke; FASD, fetal alcohol spectrum disorders; GCP, granule cell precursor; IGL, internal granular layer; IUGR, intrauterine growth restriction; ML, medial layer; NHP, non-human primate; PM, particulate matter; SGA, small for gestational age; THC, tetrahydrocannabinol.
